# Psychometric Properties of the Anxiety Measure: Stress and Anxiety to Viral Epidemics-6 (SAVE-6) for Spanish Medical Students

**DOI:** 10.3390/medicina60111803

**Published:** 2024-11-03

**Authors:** Aziz Sarhani-Robles, María Guillot-Valdés, Cristina Lendínez-Rodríguez, María Auxiliadora Robles-Bello, David Sánchez-Teruel, Nieves Valencia Naranjo

**Affiliations:** 1 Medicine Faculty, University of Granada, 18016 Granada, Spain; 2 Department of Psychology, University of Jaen, 23071 Jaen, Spainclendine@ujaen.es (C.L.-R.); nnaranjo@ujaen.es (N.V.N.); 3 Faculty of Psychology, University of Granada, 18012 Granada, Spain

**Keywords:** SAVE-6, medical students, psychometric properties, anxiety, viral epidemics, resilience

## Abstract

*Backgroud and Objective:* The aim of this study was to evaluate the psychometric properties of SAVE-6 in the medical student population and assess its gender invariance. *Subjects and Methods:* The sample consisted of 320 medical students aged 18–23 years (153 men and 167 women) who completed an anonymous online questionnaire. Data collection took place in June 2024. To assess the scale structure, a descriptive analysis of the items was carried out, followed by a confirmatory factor analysis (CFA). To analyze whether there were differences in the invariance of the measure by gender, a multigroup CFA was performed. *Results:* SAVE-6 showed high internal consistency, α = 0.89 and ω = 0.92, a minimum score of 12, a maximum score of 22, an unifactorial structure, and adequate convergent validity. Specifically, the following were found: the positive and significant relationship with HADS was 0.98 for the full scale, 0.76 for depression, and 0.91 for anxiety, and there was a negative and significant convergent validity with resilience (−0.82) and resilience to suicide attempts (−0.88). Regarding the gender invariance, relevant data is that the factor loadings between each item and the SAVE-6 factor were not the same, so women present a higher level of anxiety than men (Δχ 2 (6) = 42.53). *Discussion:* The results showed good internal reliability of SAVE-6 and good suitability. Data also revealed that they were not equal in relation to gender. Specifically, the scalar invariance revealed significant differences by items between men and women in anxiety. *Conclusions:* This scale can be applied to medical students as a reliable and valid instrument to assess the anxiety response to disease contagion in future health professionals.

## 1. Introduction

Persisting infectious diseases (e.g., malaria, HIV/AIDS, dengue, tuberculosis) and newly emerging infectious diseases in our context, such as monkeypox, Nile virus, or respiratory viruses, can cause serious and sometimes fatal diseases due to their rapid spread, raising global public health concerns [1,2,3]. Over the years, infectious disease outbreaks, such as seasonal influenza, Ebola, and SARS, have created significant challenges for health systems, affecting both the economy and, above all, the physical and mental health of the population worldwide [4,5]. In addition to widely known diseases, vector-borne diseases—those transmitted through vectors such as mosquitoes and ticks—have also become major public health concerns. These include malaria, Zika, West Nile fever, Crimean–Congo hemorrhagic fever, Chikungunya, Lyme disease, Yellow fever, Japanese encephalitis, and Rift Valley fever. The prevalence of these diseases has been on the rise in recent decades due to a combination of socio-economic factors and environmental shifts, particularly climate change and global warming, which expand the habitats of vectors and intensify transmission rates. Consequently, further research on these diseases and their public health implications is essential, particularly from the perspective of health professionals who are increasingly tasked with managing these complex and evolving threats [6]. The constant threat of new diseases continues to cause a progressive deterioration in the mental health of people who experience, among other things, fear of getting sick, concern for the health of their family members (especially the most vulnerable), and stress related to the physical and psychological consequences of infections.

In this context, healthcare workers and medical students, who are in direct contact with patients and potentially exposed to infectious agents (biological samples, contaminated surfaces in clinical settings), are particularly vulnerable to stress and anxiety [7]. Especially, the pandemic generated a high level of uncertainty due to a lack of information and fear of contagion [8]. Thus, reports show that the prevalence of anxiety, depression, and stress in Spanish university healthcare students was 21.34%, 34.19%, and 28.14% of the respondents, respectively [9].

This heightened psychological vulnerability is still evident today, with students and trainees facing ongoing challenges related to their exposure risks, which can impact their mental health and, ultimately, their professional development [10]. In particular, medical students, who are in a key training process, are affected by exposure to the risk of infection, which increases their anxiety and affects both their academic performance and long-term psychological well-being [11,12]. Poor mental health can also lead to other negative outcomes, including suicidal ideation and burnout [13]. In general, some studies indicate a gradual worsening of mental health as students advance through their medical education, which points to the urgent need for preventive and supportive measures [14,15]. In short, some medical students will experience increased depression, anxiety, interpersonal difficulties, and suicidal thoughts [16].

Given the considerable psychological burden borne by medical students, it is essential to have reliable, tailored instruments to assess their levels of anxiety, especially when exposed to the heightened risks associated with infectious diseases. However, there are relatively few tools specifically designed for this population. Some of them can be mentioned, such as the Coronavirus Anxiety Scale (CAS) by Lee [17], which focuses excessively on anxiety-related physiological arousal symptoms [17], and the Fear of COVID-19 Scale (FCV-19S) by Ahorsu et al. [18], which is reliable and valid for assessing fear of COVID-19 infection in the general population [19] but does not specifically assess anxiety.

In response to this need, the SAVE-9 (Stress and Anxiety to Viral Epidemics—9 items) scale has been developed as a valid tool to assess anxiety and work stress in health professionals. This scale, in turn, includes two factors: first, anxiety related to the epidemic, which includes six items (SAVE-6), and second, work stress related to the viral epidemic, which includes the remaining three items (SAVE-3) [17].

As the SAVE-9 scale could not be used to measure anxiety in the general population, some researchers decided to validate the SAVE-6 scale, which was found to be reliable and useful [17] and has also been validated in South Korea in cancer patients [20].

The SAVE-9 scale includes elements that are more relevant to practicing professionals rather than students in training who may experience unique stressors not directly related to professional duties. This distinction is essential, as medical students are often immersed in highly stressful, patient-facing environments without the full training or resilience strategies that seasoned professionals might have.

Despite these advances in instruments, one of the issues that remains unresolved is the specific assessment of medical students because although the SAVE-9 scale should not be applied to medical students because it covers work-related aspects, the SAVE-6 scale has not been validated for this particular population.

Why make such a distinction between healthcare professionals and medical students? There are several reasons because although they share the reality of the healthcare system, there are significant differences in how the work at hospitals in these two groups differ.

Firstly, several studies have shown that medical students often experience a range of physical and psychological distress during their formative years. As a result, their mental health has become the focus of attention, not only because of its prevalence but also because of the short- and medium-term consequences, as it has a decisive influence on the academic performance of these students [21] and its deterioration can even have a negative impact on skills essential to the practice of this profession, such as empathy, ethics, and sensitivity. In this regard, it has been found that more than 80% of medical students have experienced some degree of negative stress, and more than half have experienced various symptoms of distress, such as burnout, anxiety, poor quality of life, insomnia, depression, etc. [22].

For this reason, resilience, defined as an individual’s ability to adapt to and recover from adverse situations, has received increasing attention [23,24]. Specifically, in the medical education literature, resilience has been summarized as “the ability to withstand or cope with adversity without developing physical or psychological disability” [25]. Thus, this way of coping with life may be particularly important for medical students who have to deal with high levels of stress and pressure on a daily basis.

Resilience depends on both risk and protective factors related to individual differences, the family context, and the characteristics of the community in which a person lives. Specifically, the following elements have been described as contributing to resilience: motivation, healthy self-esteem [26], optimism, social support and attachment, staying well informed, and using distraction strategies, such as laughter and fun [27].

In terms of benefits, more resilient medical students have been found to be less likely to attempt suicide, as has been studied in some meta-analyses [28,29]; however, for these students, resilience becomes as important as the ability to be flexible in the face of adverse circumstances. This means that when faced with situations that are constantly changing and sometimes moving towards uncertain and challenging scenarios, it is important that they find appropriate strategies, regularly evaluate their effectiveness, and modify them if necessary [27].

In conclusion, medical students, like other health professionals, have to face the difficulties of performing practical activities in hospitals. However, there are no scales adapted to this group that can validly and reliably assess their level of anxiety in this type of situation of exposure to potentially dangerous viruses. For this reason, a sample of Spanish medical students was selected to confirm the hypothesis that the SAVE-6 scale follows a single-factor structural model, and we expect to find good internal consistency and convergent validity with other instruments. On the other hand, SAVE-6 is expected to be positively related to psychopathological variables and negatively related to protective psychological variables, such as low risk of suicide and resilience. SAVE-6 applied to medical students is expected to maintain the unifactorial structure of the test.

## 2. Method

### 2.1. Participants

A total of 320 people responded to a series of online questionnaires. The criteria for inclusion in this study were (1) being a medical student (2) completing all of the questionnaires, (3) living in Spain, and (4) being older than 18 years old. In short, only those participants who responded to everything asked in the questionnaires were included in the sample, so all those who did not respond to any of the items in the questionnaires were excluded.

The total sample was characterized by 153 men and 167 women between 18 and 23 years of age (M = 21.1; SD = 9.57), with no significant differences and an adequate effect size (see Table 1).

### 2.2. Instruments

#### Sociodemographic Information Including the Collection of Data on Gender and Age

Stress and Anxiety to Viral Epidemics—SAVE-6 by Chung Ahn et al. [20] is a derived subscale of SAVE-9 developed originally for measuring the stress and anxiety of the viral epidemic among healthcare workers [20]. Each of the six items is rated with a Likert 0–4 response format. The cut-off score of the SAVE-6 scale has been reported to be 15, equivalent to at least a mild degree or ≥5 on the Generalized Anxiety Disorder-7 scale. The SAVE-6 scale was found to be a reliable, valid, and useful brief measure Chung et al. 2021 [30].

The Hospital Anxiety and Depression Scale—HADS—by Zigmond and Snaith [31] in its Spanish adaptation was used [32]. This test measures anxiety (7 items) and depression (7 items) for a total of 14 items with a 0–3 point response format. In this study, the alpha for the total scale was 0.91 and 0.89 for anxiety and 0.83 for depression.

The Resilience Scale (RS-14) by Wagnild [33], specifically the Spanish version that was conducted by Sánchez-Teruel and Robles-Bello, was used [34]. The original scale measures the degree of resilience through two dimensions: personal competence, composed of eleven items, and acceptance of oneself and life, with three items. The items are presented in a seven-point Likert format, where “1 = strongly disagree” and “7 = strongly agree”. Cronbach’s alpha for the current sample is 0.94.

The Scale of Resilience to Suicide Attempts (SRSA-18) by Sánchez-Teruel et al. was used [35]. This assesses resilience to suicide attempts and predicts future suicide reattempts 6 months after the first attempt. The 18-item scale has adequate internal consistency (α = 0.88; ω = 0.89) and concurrent validity with general resilience and resilience in suicidal ideation. In the present study, its internal consistency was 0.88.

### 2.3. Procedure

The original version of SAVE-6 was extracted from the Spanish version of SAVE-9 by the same team from the link where the scale is published in Spanish. The SAVE-9 scale has been extracted from the following open-source link: https://acortar.link/3RGPq7 accessed on 20 September 2023. Only items 1, 2, 3, 4, 5, and 8 of SAVE-9 were used. Prior electronic consent was required in order for participants to be able to access this study. The bioethics committee of the first author’s university approved the research work (code JUL.22/5.LINE). Data collection was carried out in June 2024. Contacts of people studying medicine were then invited through social networks. Medical students were invited to participate in the data collection process on a voluntary basis. Once completed, the online survey was emailed to the participants, who then snowballed it to their respective contacts. Participation was free and voluntary.

The aforementioned actions were conducted via an official platform (Google Forms) provided by the university of one of the authors of this manuscript, which guaranteed the confidentiality and total anonymity of all participants. In order to proceed with the evaluation measures, it was necessary to read and accept the informed consent form in advance. Without this, access to the instruments used was not permitted. In the initial information and consent form, the contact details of the Spanish researchers, including their names and email addresses, were provided.

### 2.4. Data Analysis

Missing data accounted for less than 1% of the total. First, to assess the scale structure, a descriptive analysis of the items was performed, followed by a confirmatory factor analysis (CFA) with SPSS AMOS SPSS 23.0-IBM Corporation (Armonk, NY, USA) [36]. The method used was generalized least squares (GLSs) with bootstrap procedures for the multivariate normality test. The fit indices used were the χ^2^ index, the root mean square error of approximation (RMSEA), the comparative fit index (CFI), the Tucker–Lewis index (TLI), the normalized fit index (NFI), and the adjusted goodness-of-fit index (AGFI). The model is considered satisfactory if the RMSEA was below 0.05 and if the CFI, TLI, NFI, and AGFI were at or above 0.95 [37,38,39].

Second, we also analyzed whether there were differences in the invariance of the measure by gender using a multigroup CFA with AMOS, defining two nested models (female and male). For measurement invariance, we used the Satorra–Bentler scale (χ^2^) and its *p*-values, together with the RMSEA with a 90% CI and the CFI, as an incremental fit index [40]. Measurement invariance exists when the *p* > 0.05 of Δχ^2^ (considering sample size bias), RMSEA values are ≤0.05, and the ΔCFI value of the compared models is <0.01 [41]. A configurational invariance analysis (reference model) was conducted to test whether the groups (gender) associate the same subsets of items with the same construct. Metric invariance was also analyzed to check whether the factor loadings between each item and the factor were the same for men and women. Finally, convergent validity was measured.

## 3. Results

The results of the descriptive analysis of the items and the Kolmogorov–Smirnov test, together with the data referred to skewness and kurtosis, show moderate variability, indicating some univariate normality in this sample (Table 2). The correlation between item total and alpha showed adequate correlations for all items (>0.50), and there was no improvement in reliability if any of the items were eliminated. This suggests that all items contribute meaningfully to the overall reliability of the scale, underscoring the robustness of the SAVE-6 items in capturing consistent responses across the sample.

The data obtained showed that there was no multivariate normality in the distribution of the items (Mardia = 657.73) [42], but the goodness-of-fit indices in this sample were excellent. Specifically, this model presents an adequate χ^2^ = 187.38, df = 56, χ^2^/df (2.87; *p* < 0.01), and an RMSEA value (95% confidence interval [CI]) below 0.03 [0.02; 0.03], as well as adequate scores for the CFI (0.99), TLI (0.98) and NFI (0.97), and AGFI (0.98) values above the 0.95 limit, with a high agreement among the of-fit indices used. These fit indices suggest that the model aligns well with the observed data, reinforcing SAVE-6′s capacity to measure anxiety related to contagion effectively within this sample. According to these results, the acceptability and fit of this model to the sample of Spanish medical students is considered robust, confirming a unidimensional structure.

The configural invariance test (reference model) and the metric and scalar invariance revealed good levels of fit but also revealed that they were not equal with regard to gender. Metric invariance shows that the factor loadings between each item and the SAVE-6 factor were not the same, so women present a higher level of anxiety than men (Δχ^2^_(6)_ = 42.53; *p* < 0.06; ΔCFI = 0.02). This variance underscores the impact of gender on anxiety levels within epidemic contexts, as reflected in the differential item responses. This result is more evident in the scalar invariance that shows significant differences by items between men and women in anxiety (Δχ^2^_(7)_ = 77.61; *p* < 0.07; ΔCFI = 0.03) (see Table 3). Finally, the results referred to SAVE-6 in Spanish that we will call SAVE-6s present a high internal consistency, where α = 0.89 and ω = 0.92 in the total sample; the minimum score in this sample was 12, and the maximum score was 22. These high internal consistency values, alongside the strong factor loadings, indicate that SAVE-6s can be relied upon as a dependable measure of contagion-related anxiety across a range of severity levels in this population. And, the data for convergent validity show a positive and significant relationship (*p* < 0.01) with HADS, which was 0.98 for the full scale, 0.76 for depression, and 0.91 for anxiety, and there was a negative and significant convergent validity with resilience (RS-14 = −0.82; *p* < 0.05) and resilience to suicide attempts (SRSA-18 = −0.88; *p* < 0.05). These convergent validity results emphasize SAVE-6s’ strength as an anxiety measure by its significant association with well-established scales for depression and anxiety, as well as its inverse relationship with resilience measures, providing support for its clinical relevance in high-stress educational settings.

## 4. Discussion

The aim of this study was to examine the psychometric properties of the SAVE-6 scale in a sample of medical students. Our results indicated good internal reliability of SAVE-6 and good suitability for factor analysis. Factor analysis showed a unidimensional structure of SAVE-6 in Spanish that explained 88.10% of the variance. The CFA and additional multigroup CFAs revealed a good fit of the indices. The SAVE-6 scores correlated well with the HADS, indicating good convergent validity. The scale has considerable value in measuring the severity of the two most common psychiatric symptoms in the general population, namely, anxiety and depression in this situation.

It has been suggested that there is a relationship between anxiety and depression with gender, so it is still interesting to continue to provide empirical evidence in this regard [43]. This relationship is further complicated if age is also taken into account. Some studies indicate that anxiety symptoms (state anxiety and trait anxiety) are known to be more common in women in early life [44], but their incidence decreases progressively with age. Among the possible causes that have been suggested for this reality are female biological factors, such as hormonal functioning, neural circuitry, and neurotransmitter activity, which cause women to approach life differently from men and to respond differently to different stressful situations, even predisposing them to experience anxiety more frequently [22].

Other studies have suggested that gender may be a factor in an individual’s response to the traumatic and stressful situation posed by high-risk infectious diseases or when the pathology becomes epidemic, with women reporting more psychological effects, especially a greater fear of contagion, perception of personal risk, fear of dying, and fear of infection than men [45]. This may be due to both physical and cultural factors, as women express their emotions more easily (it is culturally acceptable) and, therefore, seek help more quickly than men.

On the other hand, some research also suggests that resilience tends to increase with age [46], which involves a learning process on the part of the individual as he or she copes with stressful life situations and often requires intentionality on the part of those educating him or her at different stages of life. Thus, as resilience strengthens over time, it acts as a significant buffer against both chronic and acute stressors, highlighting the need for longitudinal support mechanisms that foster resilience throughout medical education and beyond. By fostering resilience early, particularly in stressful learning environments, educators can equip students with tools for coping that are essential for a sustainable and fulfilling career in medicine. Particularly, in view of the psychological distress suffered by medical students (aggravated in recent years by the pandemic) as a result of the difficult experiences they encounter on a daily basis, it is essential that medical school curricula explicitly include emotional training and, in particular, resilience strategies, so that these professionals in training can carry out their work with the highest possible quality of life, which in turn leads to careful and excellent care for patients. In this sense, resilience would play the role of mediator or protector between external stressors, students’ personal characteristics, and their mental health [22].

Additionally, given the significant negative relationship observed between resilience and suicidality, these results underline the protective role of resilience against suicide risk [47]. This relationship indicates that higher levels of resilience are associated with a lower tendency to experience suicidal thoughts, which is particularly relevant in vulnerable populations, such as medical students, who face high levels of stress and psychological distress [48,49]. Consistent with these findings, incorporating resilience-building strategies into medical students’ training programs could reduce their vulnerability to suicidal ideation and enhance their ability to cope with adversity. The integration of these protective factors not only contributes to students’ emotional well-being but also has the potential to minimize the risk of suicidal behavior in high-stress environments [50,51].

Furthermore, resilience-building interventions could contribute to a broader cultural shift in medical training environments, where mental health becomes a prioritized aspect of professional development rather than an afterthought. Such approaches could include workshops, peer support groups, mentorship programs, and other resources aimed at promoting resilience and reducing burnout. By implementing a proactive approach to mental health within medical curricula, institutions can better address both the immediate and long-term psychological needs of medical students, ultimately supporting a healthier future workforce.

Given the diversity of the results, which are probably associated with the contribution of different factors (e.g., the presence of previous disorders, age or gender of the participants), SAVE-6 offers an instrument adapted to the Spanish population that allows mental health professionals to make more adjusted and individualized decisions, especially when levels of generalized anxiety stand out as a relevant alteration in mental health status in infectious disease risk situations.

With only six items, SAVE-6 is a brief tool that facilitates a quick assessment without overburdening health workers. This is especially useful in high-pressure medical environments where time is limited. As a quick and specific tool, SAVE-6 allows for rapid identification of health workers who are experiencing high levels of anxiety and stress related to possible infectious infections. This enables preventive or early intervention measures to be taken, such as psychological support or adjustment of working conditions.

This study is not without limitations. First, the information was extracted through self-reports and could not be compared with other sources of information on the mental state or possible mental disorders of the participants. Secondly, the sample could have been extended to other mental health workers, such as nurses, or medical students in other settings and contexts, such as rural areas.

Finally, it should be noted that while SAVE-6 offers a practical and accessible measure of anxiety related to infectious disease risk, future research could benefit from exploring its applicability across diverse healthcare populations and varying contexts of exposure. Such studies could further validate its utility as a global tool for assessing epidemic-related anxiety and potentially inform interventions across a broader array of healthcare environments.

In conclusion, the SAVE-6 scale could be applied to medical students, as it has been shown to be a reliable and valid instrument for assessing anxiety response to disease contagion. The SAVE-6 scale’s demonstrated reliability and unidimensionality not only suggest that it is a robust measure of epidemic-related anxiety but also highlight its potential utility as an accessible screening tool within clinical and academic settings. Its application can facilitate early interventions that mitigate stress and anxiety among medical trainees, helping to support their overall mental health and academic success.

## Figures and Tables

**Table 1:** Description of sociodemographic data.

	N (%)	Chi-Square	d.f.	Phi
Gender		1.49 ^ns^	1	0.61
Women	167			
Men	153			
Age		2.33 ^ns^	1	0.72
18–20	141			
21–23	179			
Total	320			

ns = no significant; d.f. = degree of freedom; Phi = effect size.

**Table 2:** Descriptive statistics and analysis of the SAVE-6 item.

Item	M (SD)	K-S	A	C		
			ET = 0.37	ET = 0.18	*r* Item-Total	α if Item Deleted
1	2.8 (2.12)	0.80 *	−1.26	−2.22	0.79	0.61
2	2.62 (2.39)	0.71 ^ns^	0.57	0.54	0.67	0.82
3	2.58 (2.72)	0.70 **	1.22	1.11	0.78	0.80
4	2.84 (1.01)	0.76 *	−0.34	−0.85	0.60	0.79
5	2.17 (2.20)	0.79 ^ns^	0.47	0.79	0.71	0.73
6	12.04 (2.75)	0.70 ^ns^	0.46	0.93	62	0.81
Total	14.49 (5.25)	1.21 *	−0.71	0.72	1	0.89

M = mean; SD = standard deviation; S = skewness; K = kurtosis; SE = standard error of skewness and kurtosis; K-S = Kolmogorov–Smirnov test; * significant correlation at the 0.05 level (bilateral); ** significant correlation at the 0.01 level (bilateral); ns = non-significant.

**Table 3:** Fit indices for the invariance test in gender and age.

	χ^2^	df	p	RMSEA (95%CI)	CFI	Δχ^2^	ΔCFI
Men (*n* = 153)	31.73	25	0.02	0.03 [0.01; 0.04]	0.95		
Women (n = 167)	42.2553	25	0.00	0.02 [0.01; 0.03]	0.98		
Configural invariance gender	77.12	43	0.71	0.02 [0.011; 0.031]	0.96		
Metric invariance gender	89.16	45	0.22	0.03 [0.032; 0.043]	0.96	37.61 * (Δdf = 6)	0.02
Scalar invariance gender	105.11	55	0.34	0.03 [0.021; 0.047]	0.98	42.53 * (Δdf = 7)	0.03

χ*^2^* = chi-square; df = degree of freedom, *p* = significance level; RMSEA = root mean square error of approximation; CFI = comparative fit index; Δχ^2^ = difference test between the configural and metric or scalar invariance models; ΔCFI = difference test between the comparative fit index; * = *p* < 0.05; ns = not significant.

## Data Availability

The data presented in this study are available upon request from the corresponding author. (please specify the reason for restriction, e.g., the data are not publicly available due to privacy or ethical restrictions).
